# The Past, Present, and Future of Psychotherapy Manuals: Protocol for a Scoping Review

**DOI:** 10.2196/47708

**Published:** 2023-06-30

**Authors:** Katherine Wislocki, Mai-Lan Tran, Emily Petti, Rosa Hernandez-Ramos, David Cenkner, Miranda Bridgwater, Ghazal Naderi, Leslie Walker, Alyson K Zalta

**Affiliations:** 1 Department of Psychological Science University of California, Irvine Irvine, CA United States

**Keywords:** psychotherapy, manual, protocol, primer, evidence-based treatment, scoping review, review methodology, mental health, scoping, manual, guideline, guidelines, book based, manuals, knowledge translation

## Abstract

**Background:**

Psychotherapy manuals are critical to the dissemination of psychotherapy treatments. Psychotherapy manuals typically serve several purposes, including, but not limited to, establishing new psychotherapy treatments, training providers, disseminating treatments to those who deliver them, and providing guidelines to deliver treatments with fidelity. Yet, the proliferation of psychotherapy manuals has not been well-understood, and no work has aimed to assess or review the existing landscape of psychotherapy manuals. Little is known about the breadth, scope, and foci of extant psychotherapy manuals.

**Objective:**

This scoping review aims to identify and explore the landscape of existing book-based psychotherapy manuals. This review aims to specify the defining characteristics (ie, foci, clinical populations, clinical targets, treatment type, treatment modality, and adaptations) of existing book-based psychotherapy manuals. Further, this review will demonstrate how this information, and psychotherapy manuals more broadly, has changed over time. This project aims to make a novel contribution that will have critical implications for current methods of developing, aggregating, synthesizing, and translating knowledge about psychotherapeutic treatments.

**Methods:**

This scoping review will review book-based psychotherapy manuals published from 1950 to 2022.This scoping review will be informed by guidance from the Joanna Briggs Institute Scoping Review Methodology Group and prior scoping reviews. Traditional search and application programming interface–based search methods will be used with search terms defined a priori to identify relevant results using 3 large book databases: Google Books, WorldCat, and PsycINFO. This review will leverage machine learning methods to enhance and expedite the screening process. Primary screening of results will be conducted by at least 2 authors. Data will be extracted and double-coded by research assistants using an iteratively defined codebook.

**Results:**

The search process produced 78,600 results, which were then iteratively deduplicated. Following deduplication, 50,583 results remained. The scoping review is expected to identify common elements of psychotherapy manuals, establish how the foci and content of manuals have changed over time, and illustrate coverage and gaps in the landscape of psychotherapy manuals. Results from this scoping review will be critical for future work focused on developing, aggregating, synthesizing, and disseminating knowledge about psychotherapeutic treatments.

**Conclusions:**

This review will provide knowledge about the vast landscape of psychotherapy manuals that exist. Findings from this study will inform future efforts to develop, aggregate, synthesize, and translate knowledge about psychotherapeutic treatments.

**International Registered Report Identifier (IRRID):**

DERR1-10.2196/47708

## Introduction

Psychologists have long disseminated therapeutic knowledge and procedures through writings such as books and papers. For example, Freud first published *The Interpretation of Dreams* in 1899. However, the origins of the psychotherapy manual are unclear, and what qualifies as the first psychotherapy manual has not been well-established. Previous work has endorsed different publications as the first psychotherapy manual, including Wolpe’s [1] 1958 *Psychotherapy by Reciprocal Inhibition*, Kelerman and Neu’s [2] 1973 (unpublished) *Interpersonal Approach to Treating Depression*, Paul’s [3] 1966 *Insight vs. Desensitization in Psychotherapy*, and Beck’s [4] 1979 *Cognitive Therapy for Depression*. Though the origins of manuals are not well-understood, psychotherapy manuals have evolved to play an important and prominent role in clinical research, education, and practice.

Since their emergence, the value of psychotherapy manuals for the field has been widely and contentiously debated, receiving both praise and criticism [5-11]. Critics have argued against the use of manuals for being prescriptive, difficult to apply to real-world clinical practice, antagonistic to clinician intuition, harmful to therapeutic alliance, and detrimental to the impactful delivery of therapeutic skills and techniques [5,6]. By contrast, proponents highlight the numerous functions that psychotherapy manuals serve. First, they are essential for conducting research and establishing evidence-based psychotherapeutic treatments as they provide a protocol for treatments to test and evaluate treatment efficacy [2,7-11]. Second, creation of a manual legitimizes new and emerging psychotherapy treatments, such that without a manual, it is difficult to disseminate new treatments to practicing clinicians [9-12]. Third, manuals provide a structure that can be used to understand and evaluate fidelity or adherence to treatment [2,7,9,10]. Fourth, manuals aid in the training and development of clinicians [2,7-10,13]. Throughout their careers, clinicians can use manuals to learn new practices or improve their own practice. Indeed, research has indicated that practicing clinicians have read multiple manuals [14]. Finally, manuals are used to communicate information about treatments and enhance the legitimacy of psychotherapy practice to outside stakeholders (ie, policymakers and consumers). Because of these benefits, manuals have become the primary method of disseminating psychotherapeutic treatments, and their proliferation has been extensive over the past half-century [11].

Despite the prominence of manuals in psychotherapy research and practice, what constitutes a psychotherapy manual is not well-defined [15]. Clarity about what defines psychotherapy manuals is also mixed among those who use them, as demonstrated through empirical research with clinicians [7,14]. Previous works have attempted to define the psychotherapy manual and its necessary components [2]. Common components include a theoretical basis for treatment, a historical overview of treatment, a guide for delivering treatment, an overview of information about delivering treatment, troubleshooting information, patient or client dialogue, case or vignette examples of treatment, and supporting resources for delivering treatment (eg, assessment tools, fidelity checklists, client worksheets, case vignettes, and session outlines) [7,14]. The form of psychotherapy manuals is also not strictly defined, with “psychotherapy manuals” being disseminated as multiple forms, including books, individual book chapters, journal papers, gray literature, theses or dissertations, government publications, and more. However, books remain a popular method for disseminating psychotherapy manuals.

Given that psychotherapy manuals have become the predominant method for disseminating psychotherapy techniques outside of direct face-to-face supervision, a review of existing book-based manuals is critical for understanding the evolution in foci and content of manuals over time, the elements of psychotherapy manuals, and the current coverage and gaps of specific topics in extant psychotherapy manuals. A scoping review is the optimal approach to review the landscape of existing psychotherapy manuals. Scoping reviews focus on mapping existing knowledge on a topic, and they can be particularly advantageous when all relevant information on a topic is infeasible or impossible to attain. Given the decentralization and unknown breadth of psychotherapy manuals, a scoping review is the ideal design for understanding this landscape and addressing this problem [16].

Although scoping reviews have typically relied on manual search, screening, and data extraction procedures, machine learning has increasingly been used to support the review process [17]. Conducting reviews is resource-intensive [17], and as the body of literature gets larger, the labor associated with conducting reviews increases. Previous work has illustrated that the disparity in size between search results and the final sample is vast, with an average inclusion rate of ~3% of search results [18]. To address this problem, machine learning has been used to efficiently and effectively aid the review process [17]. Previous work has illustrated the use of machine learning techniques in the search, title or abstract screening, full-text screening, and information extraction phases [19]. While rapidly expanding, current machine learning techniques are only recommended for certain phases of the process, including, particularly, the screening phase [19,20]. Review platforms themselves have also increasingly leveraged machine learning components within their own software [20-22].

Leveraging machine learning techniques, this scoping review will be the first to perform a high-level review of book-based psychotherapy manuals. Currently, there is no systematic or even quasi-systematic knowledge about the psychotherapy manuals that exist. Given the large role of psychotherapy manuals in training, practice, and research in clinical psychology, this is a critical gap in knowledge that this scoping review will fill. This review will address several aims. Primarily, this review will aggregate and synthesize information about a large portion of manuals that exist. Within this aim, this review will specify the foci, clinical populations, clinical targets, treatment type, treatment modality, and adaptations of existing manuals. Further, this review will demonstrate how psychotherapy manuals have changed over time. The findings from this review will also support future efforts to develop, aggregate, synthesize, and translate knowledge about psychotherapeutic treatments. This paper delineates the protocol and methods used to address these aims.

## Methods

### Study Design

This scoping review will be informed by guidance from the Joanna Briggs Institute Scoping Review Methodology Group [23] and previous literature on scoping reviews [20]. Specifically, this scoping review will use a modified form of the scoping review framework outlined by Peters and colleagues [16]. Slight modifications to this framework will be necessary to capture information specific to psychotherapy manuals in book form.

### Search Strategy

Search terms and search strategy were developed collaboratively with the research team and subject matter librarians. The full search approach can be found in Multimedia Appendix 1. PsycINFO, Google Books, and WorldCat were searched for this review, searched in July 2022. These databases were selected based on (1) catalog size, (2) availability of relevant data for review, and (3) feedback from study authors and subject matter librarians. Some traditional scholarly databases, such as PubMed, do not consistently index psychotherapy books and thus were not used for this review. For PsycINFO, traditional database search methods were used. A search statement was developed for PsycINFO that combined both treatment and format language. Search terms for PsycINFO consist of “(psychotherap* OR therap* OR clinic* OR treat* OR interven* OR practi* OR counsel*) AND (manual OR protocol OR guide OR handbook OR primer).” PsycINFO offers over 5 million bibliographic records across numerous disciplines [24]. Search terms for PsycINFO included the aforementioned search statement as well as additional terms to include books but exclude book chapters (see Multimedia Appendix 1). Application programming interfaces (APIs) were used to search Google Books and WorldCat. As of October 2019, Google Books maintained a catalog of over 40 million books [25]. As of November 2022, WorldCat maintained a catalog of over 542 million bibliographic records [26]. APIs allow for the large-scale aggregation of search results that is unavailable through other methods. Compound search terms were created from the predefined search statement for the Google Books API. More specifically, permutations were created from terms from both sides of the search statement’s “AND” to create briefer search terms (ie, “therap* manual,” “therap* handbook,” “clinic* primer,” and “clinic* protocol”) that were more feasible for API search methods (see Multimedia Appendix 1). Search strategy for WorldCat leveraged specific subject headings for the search process (see Multimedia Appendix 1). WorldCat search procedures were conducted by subject matter librarians from the University of California, Irvine. All initial search processes for this review were conducted in July 2022. Additional search processes will occur following the coding phase and be based on included results. This additional search process will be a snowball search based on bibliometric information of included results. Data from API queries were extracted and formatted to citations.

Deduplication was conducted in Python through an iterative duplication process. Deduplication focused on deduplicating by multiple parameters, given that some results may have the same titles (ie, books of different editions) or authors. Multiple rounds of deduplication were performed. First, deduplication by URL was performed to ensure that equivalent results from the same source were removed. Next, deduplication by ISBN identifiers was performed to ensure that duplicate results based on ISBN-10 or ISBN-13 numbers were removed. Finally, deduplication by a combination of title, description, AND year was performed, as well as a combination of title, author, AND year. Manual deduplication will be conducted throughout the later review phases, if necessary. Citations will be managed in reference management software.

### Identification and Screening

Inclusion criteria were developed by study authors and subject matter librarians. First, manuals must be in the English language. Second, manuals must be a published book as this review focuses on books (ie, print books and electronic books), rather than other publication forms (ie, unpublished manuscripts, book chapters, journal papers, and research documents). Third, manuals must have been published between 1950 and 2022, as this reflects a broad time period that includes the creation of a vast majority of modern psychotherapies (ie, cognitive behavioral therapy and rational emotive behavior therapy). Additionally, this encompasses the time period in which previously identified “first” manuals were published [1-4]. Fourth, manuals must consist of a majority of instructional content about how to deliver a psychotherapeutic treatment or treatment components. This is defined as at least 50% or more content dedicated to instruction about delivering techniques or interventions. In cases where this is unclear, other resources may be used to assist with verification, such as book previews and freely available copies of books. Screening decisions will be made based on the information provided through titles or descriptions. After screening decisions have been completed, trained coders will code each included resource. For the coding process, more information may be sought through identified sources (ie, scholarly book reviews, publisher websites, and other book databases). If the additional information referenced during the coding process reveals that a manual should have been excluded, the manual will be excluded at this stage. This information may result in included resources being excluded during the coding process. Full information regarding inclusion or exclusion decisions at each stage will be outlined in the subsequent scoping review [16]. Fifth, manuals must be intended for use by a mental health professional (ie, psychologist, social worker, counselor, nurse, or physician). While some manuals have been previously addressed to consumers or lay providers, this review opts to focus on manuals that can be used by professionals to deliver mental health care. Results were excluded if they were a thesis or dissertation, another form of gray literature, or did not meet established inclusion criteria.

Screening of book descriptions and titles will be conducted in Rayyan (Rayyan Systems) [21]. Due to the size of this review, Rayyan’s machine learning enhanced ranking system will be used as a form of active learning. Rayyan uses a support vector machine prediction system based on previous inclusion or exclusion decisions and text features (ie, unigrams and bigrams) [21]. Following an initial introductory period where eligibility screening is conducted by the research team, Rayyan’s ranking system will gradually “learn” the parameters (ie, lexical patterns in titles and descriptions) of results that are receiving certain decisions (ie, “included” and “excluded”). Rankings of results with no decision will be regenerated by KW on a regular basis throughout the screening process. Results will be screened in accordance with this ranking system, with highly ranked results (ie, those most likely to be included) prioritized for screening. Approximately 15%-20% of all results will be screened by KW and a member of the screening team. The remaining records will be screened for inclusion by machine learning enhanced inclusion or exclusion and supplemented input by KW, mirroring previous work and recommendations [20-22].

Screening fidelity (ie, 80% agreement) will be met through multiple rounds of test screening across the screening team and AZ. Results that will be screened by the screening team will receive 2 decisions, 1 made by KW and 1 made by another author. Screening decisions will be made on the basis of information provided (ie, book title and book description). All disagreements will be resolved through consensus discussions between screeners for each result in conflict.

### Coding Process

A codebook will be devised by input from all authors. An initial codebook will be created by KW and AZ. Feedback on the codebook will be solicited from the screening team to ensure that codes reflect relevant and extractable information from results. Additionally, the codebook will be further iteratively defined through the test coding process, with additional codes proposed, discussed, and agreed upon by the coding team, KW, and AZ. Coding will be performed for all included manuals. Fidelity will be established through 3 rounds of test coding prior to the beginning of coding. The coding team will code each result based on the information provided through bibliometric information (ie, book title and book description) as well as open-access information such as scholarly book reviews, freely available published portions of the book, and information provided via official sellers (ie, publisher websites, Amazon, and scholarly databases). Information outside of bibliometric data will be used when coding cannot be adequately performed on the information provided. Scholarly databases will be used to search book reviews when more information is necessary for coding purposes. Code disagreements will be addressed through consensus discussion or adjudication of disagreements by KW.

The initial codebook includes bibliometric information (ie, title, author, publisher, and date published), edition number, clinical target, clinical population, treatment name, treatment orientation, treatment notes, mental health provider target, treatment delivery setting, type of treatment (ie, individual, group, couples, family, and parent-child), age of treatment target, as well as dichotomous codes for several potential adaptations to treatment based on gender identity, race or ethnicity, culture, physical illness or disability, religion, employment or role, and other adaptations that are not covered by previous categories.

## Results

This scoping review is currently being conducted. Initial search across all sources was completed in July 2022. The search process produced 78,600 results, which were then iteratively deduplicated. Following deduplication, 50,583 results remained. Nearly 14.2% of results (n=7171) have been screened. A portion of included results (n=775) have been coded. Full results from this review will be submitted for publication in peer-reviewed journals.

## Discussion

### Principal Results

The proposed review will be the first to synthesize information about the existing landscape of psychotherapy manuals with the goal of addressing critical and unanswered questions. More specifically, we aim to identify the features of existing manuals (ie, foci, clinical populations, clinical targets, treatment type, treatment modality, and adaptations), illuminate the ways in which psychotherapy manuals have evolved over time, and highlight current resource allocation and gaps evidenced by existing manuals. The findings from this review will be critical for future efforts to develop, aggregate, synthesize, and translate knowledge about psychotherapeutic treatments.

### Comparison With Prior Work

Prior work that has focused on reviewing psychotherapy manuals has been narrowly limited to a specific treatment type, treatment focus (ie, eating disorders) [27], or manual format (ie, manuals from randomized controlled trials) [28]. This work has been impactful for the field, but critical gaps remain as to the broader landscape and evolution of psychotherapy manuals. Digital methods have become increasingly important for conducting research across myriad disciplines [29]. Past scholarship in digital humanities and digital history has called for the use of digital technologies to supplement psychology research, particularly in the history of psychology [29], and this scoping review aims to build on this work.

### Limitations

There are several limitations to this work. Psychotherapy manuals can be disseminated through myriad forms and methods. Because of the disparities in how psychotherapy manuals are published and disseminated, the ability to systematically aggregate all knowledge related to this topic is arguably impossible. Therefore, we limited the search strategy to books to capture the predominant method by which manuals are disseminated. This will exclude psychotherapy manuals disseminated through peer-reviewed papers and gray literature, for example.

Certain books used as psychotherapy manuals may not meet our inclusion criteria. For example, providers have previously used client-facing (ie, “self-help”) books as psychotherapy manuals, yet these resources will be excluded from this review. This was decided by the authors to limit the inclusion of materials that are not focused on providing instruction to mental health professionals about treatments and treatment delivery. Additionally, providers may use other types of books or resources to inform their clinical practice that do not focus directly on delivery of an intervention (eg, books focused on how to improve 1 specific technique within a treatment such as assigning homework, books on how to practice culturally competent care across many treatment modalities for specific populations, and books focused on developing therapeutic alliance across modalities). While these resources may be beneficial to delivery of psychotherapeutic treatments, they do not focus on instructing mental health professionals on how to deliver treatment.

Given the history of psychotherapy manuals and psychotherapy more generally, this scoping review aims to be informed by the changing definitions and ontologies in psychotherapy research. However, it is likely that through this scoping review, a modern qualitative coding may not encompass the changing definitions of psychotherapy treatments and the language used to describe them. Coding will be performed based on the bibliometric data, which is inherently reflective of the contemporary definitions in which a manual was produced. For example, the changing understanding of “behaviorism” and “behavioral treatments,” as well as what constitutes “cognitive therapy” then versus now, may not be completely understood through the results from this review.

It is possible that the databases used within this review may not have a comprehensive list of psychotherapy manuals; however, the selected databases have extensive and growing catalogs [24-26]. Data quality may also differ widely within large databases. For some results, full information may be available, others may have gaps in information provided. Further, database entries may not be fully accurate, as some databases allow for manual entry of book information. Because of this, included references will be cross-checked with other sources (ie, scholarly book reviews, other databases, and publisher information), when possible. Finally, leveraging machine learning to expedite the screening process may result in increased false positives (ie, manuals that were included that should not have been included) and false negatives (ie, manuals that were excluded that should not have been excluded) [30]. Previous work using machine learning in title or abstract screening has demonstrated strong performance in not only accelerating the screening process but also doing so with high accuracy when combining with individual review [20,30].

### Conclusions

Notably, this review will have numerous strengths. Although this is a scoping review, significant efforts will be taken to ensure that a wide variety of manuals will be included, increasing the potential representativeness of the results. Further, the use of these novel methods within this review will expand upon the growing literature on the use of machine learning to enhance reviews [17]. Most importantly, to our knowledge, this is the first large-scale review of book-based psychotherapy manuals. Thus, the benefits and implications of this work will be far-reaching for the development, dissemination, and implementation of psychotherapy knowledge. Given that a review of this topic and focus has yet to be done, this study will provide necessary information for the field’s understanding of the landscape of psychotherapy manuals. Accomplishing this will not only provide critical information on the evolution of the treatment manual, but also how treatment manuals may be improved to meet the needs of users going forward. Previous work has indicated that use of manuals may vary across different types of users [14,31]. This review has particular use in identifying current resource allocation and extant gaps, as well as how this has shifted over time. With this information, future work will be able to better address those gaps, synthesize treatment-specific knowledge, and engage in the development of more effective methods of dissemination of psychotherapy.

## Appendix

Multimedia Appendix 1 Search approach.

## Abbreviations

API: application programming interface

## Data Availability

The data sets generated during and/or analyzed during this study are available from the corresponding author on reasonable request.
