# Supplementary material for: The Past, Present, and Future of Psychotherapy Manuals: Protocol for a Scoping Review
Source: JMIR Res Protoc. 2023 Jun 30;12:e47708. doi: 10.2196/47708 (PMC10365618; doi:10.2196/47708)
Supplement: Multimedia Appendix 1 [file resprot_v12i1e47708_app1.docx]

**Search Strategy**

**PsycINFO**

Platform: PsycINFO

Search ran: July 2022

13,570 results

(psychotherap* OR therap* OR clinic* OR treat* OR interven* OR practi* OR counsel*) AND (manual OR protocol OR guide OR handbook OR primer) AND DTYPE(Books) AND NOT DTYPE(Chapter)

**WorldCat**

Platform: WorldCat Search API

Search ran: July 2022

42,918 results

**Queried terms (formatted for API requirements):**

sub=Therapy-- Handbooks, manuals, etc.

sub=Psychotherapy-- Handbooks, manuals, etc.

sub=Psychopathology-- Handbooks, manuals, etc.

sub=Disorders-- Handbooks, manuals, etc.

sub=Psychotherapy-- methods

sub=Mental illness Treatment

sub=Psychotherapy-- General

(Search Notes: Search terms were queried separately. Search was performed by subject matter librarians with API credentials.)

**Google Books:**

Platform: Google Books Search API

Search ran: July 2022

22,112 results

**API base query:** "https://www.googleapis.com/books/v1/volumes?q={}&printType=books&maxResults={}&startIndex={}”

**Queried terms (formatted for API requirements):**

'psychotherap%20manual'

'psychotherap%20protocol'

'psychotherap%20guide'

'psychotherap%20handbook'

'psychotherap%20primer'

'therap%20manual'

'therap%20protocol'

'therap%20guide'

'therap%20handbook'

'therap%20primer'

'clinic%20manual',

'clinic%20protocol'

'clinic%20guide'

'clinic%20handbook'

'clinic%20primer'

'treat%20manual'

'treat%20protocol',

'treat%20guide',

'treat%20handbook'

'treat%20primer'

'interven%20manual'

'interven%20protocol'

'interven%20guide',

'interven%20handbook'

'interven%20primer'

'practi%20manual',

'practi%20protocol'

'practi%20guide'

'practi%20handbook'

'practi%20primer'

'counsel%20manual'

'counsel%20protocol'

'counsel%20guide'

'counsel%20handbook'

'counsel%20primer'

(Search Notes: Compound search terms were created from the original search list and formatted to meet API search requirements. Each compound term was searched separately. Pagination through the returned results was done incrementally through maxResults and startIndex functions within the query.)
